# From Localized Scleroderma to Systemic Sclerosis: Coexistence or Possible Evolution

**DOI:** 10.1155/2018/1284687

**Published:** 2018-01-30

**Authors:** Giuggioli Dilia, Colaci Michele, Cocchiara Emanuele, Spinella Amelia, Lumetti Federica, Ferri Clodoveo

**Affiliations:** Scleroderma Unit, Chair of Rheumatology, University of Modena and Reggio Emilia, Modena, Italy

## Abstract

**Background:**

Systemic sclerosis (SSc) and localized scleroderma (LoS) are two different diseases that may share some features. We evaluated the relationship between SSc and LoS in our case series of SSc patients.

**Methods:**

We analysed the clinical records of 330 SSc patients, in order to find the eventual occurrence of both the two diseases.

**Results:**

Eight (2.4%) female patients presented both the two diagnoses in their clinical histories. Six developed LoS prior to SSc; in 4/6 cases, the presence of autoantibodies was observed before SSc diagnosis. Overall, the median time interval between LoS and SSc diagnosis was 18 (range 0–156) months.

**Conclusions:**

LoS and SSc are two distinct clinical entities that may coexist. Moreover, as anecdotally reported in pediatric populations, we suggested the possible development of SSc in adult patients with LoS, particularly in presence of Raynaud's phenomenon or antinuclear antibodies before the SSc onset.

## 1. Introduction

Systemic sclerosis (SSc) is a connective tissue disease characterized by different degrees of skin fibrosis and visceral organ involvement. The etiology of SSc remains obscure; the disease appears to be the result of a multistep and multifactorial process, including immune system alterations, under the influence of genetic and exogenous (toxic or infectious) factors [1].

Morphea, also known as localized scleroderma (LoS), is a distinctive inflammatory disease involving the skin and the subcutaneous tissue, characterized by excessive collagen deposition that ultimately leads to fibrosis. Differently from SSc, Raynaud's phenomenon, typical autoantibodies, and visceral involvement are generally absent.

The incidence of LoS is around 0.3 to 3 cases per 100.000 inhabitants/year [2]. It affects commonly Caucasian women, with a women/men ratio of 2–4/1, a similar prevalence in children and adults with a peak in the fifth decade of life in adults, whereas 90% of children are diagnosed between 2 and 14 years of age [3, 4].

Etiology of LoS is unknown, even if the probable trigger is a vascular injury that culminates in increased collagen production and decreased collagen destruction [5].

Plaque morphea lesions have an initial inflammatory (or active) stage of erythematous to lilaceous dusky patches or plaques; over time, the center becomes white and sclerotic, and the borders take on a characteristic “lilaceous ring.” When the active stage ends, white sclerotic plaques with postinflammatory hyperpigmentation may be found. LoS is classified according to clinical presentation: the most widely used classification in literature is the “Mayo Clinic Classification” [6, 7].

Plaque morphea is the most common presentation in adults, unlike the linear morphea that is more common in children and it often presents with fibrosis of underlying tissues up to bone. The subcutaneous tissue and the muscular fascia are targeted by the deep morphea. Finally, the generalized and the bullous morphea are rare clinical entities [3].

Though LoS is known as a dermatologic disease, it has also been reported in literature the possibility of visceral involvement, in the case of overlap with other autoimmune diseases or as possible evolution towards a systemic form; the latter possibility was described anecdotally in pediatric cases [8, 9].

Despite distinct clinical entities, SSc and LoS present analogue histopathological findings [3–5]; furthermore, the presence of autoantibodies or Raynaud's phenomenon (RP) could be reported also in LoS [3]. In this perspective they might represent two extremities of the same spectrum of disease.

The aim of our study was to retrospectively evaluate a large SSc cohort in order to investigate the relationship between SSc and LoS.

## 2. Patients and Methods

We retrospectively studied 330 patients fulfilling the ACR/EULAR criteria for SSc [10] referring to our university-based Rheumatology Unit from January 2003 to July 2017.

The eventual coexistence of LoS and SSc diagnosis was searched for each patient in the medical records. The clinical, laboratory, and instrumental features were available for all patients, from the first visit at our referral center and throughout the follow-up. In every patient, the description of cutaneous sclerosis was registered, for the purpose of an early SSc diagnosis or to document the progression of the cutaneous sclerosis.

In case of LoS, the lesions were described as regards number, site of localization, macroscopic aspects, and histological features obtained by skin biopsy, which is routinely prescribed for new patients. In these cases, the referral to our center was indicated by the dermatologist who first evaluated the subjects.

Systemic symptoms and signs evocative for SSc, such as presence of RP or acrocyanosis, telangiectasias or calcinosis, visceral involvement such as interstitial lung disease, or esophagus dyskinesia were always reported. Skin disorders different from morphea were also included in the records.

Laboratory blood tests, including erythrocyte sedimentation rate, c-reactive protein, blood cell counts, liver, kidney, and thyroid function assessments, were routinely registered. Moreover, spirometry, lung diffusion for carbon monoxide test, chest high-resolution computed tomography, echocardiography, nailfold videocapillaroscopy, and esophagus X-ray were carried out in all patients at the baseline and during the follow-up, according to patients' clinical conditions.

Possible exogenous toxic agents, such as cigarettes smoking, occupational and environmental exposures, and the eventual presence of comorbidities, were reported.

Finally, therapies administered for both localized and systemic scleroderma were registered.

## 3. Results

In total, 8/330 (2.4%) SSc patients presented also LoS (Table 1). Six SSc female patients (1.8%) had a clinical history of LoS prior to SSc diagnosis (all limited SSc subtype). The mean age at the time of LoS onset in these 6 cases was 43.5 years and the median time interval between Los and SSc diagnosis was 18 (range 0–156) months.

Other 2 SSc patients (50 F, 70 F) developed LoS after 5 and 10 years of follow-up, at the trunk and left pretibial area, respectively; both patients were anticentromere positive and with limited skin SSc subset.

Skin biopsies confirmed the diagnosis of LoS, showing nonspecific inflammatory infiltrate, collagen fiber deposition, and dermis sclerosis.

In the 6 patients with LoS before SSc, RP preceded LoS in 2 cases of 48 and 4 months, respectively; in the remaining 4 patients RP occurred after LoS onset, along with other SSc systemic symptoms.

Cutaneous involvement was represented by patches of skin sclerosis localized in limbs, trunk, or face; in one case linear LoS was reported. A single lesion was found in 3/6 patients, while the remaining cases presented multiple lesions.

Cutaneous limited SSc was diagnosed in all patients. During the follow-up, 4/8 patients developed digital ulcers (pitting scars and ulcers on calcinosis), 4/8 esophagopathy confirmed with barium swallow test and only 1/8 interstitial lung disease. No cardiac or renal involvements were reported; moreover, 5/8 patients complained arthromyalgias in absence of arthritis or myositis.

All patients underwent a nailfold videocapillaroscopy test evidencing a typical SSc pattern [11] in 6/8 patients (active pattern in 3, early pattern in 3 cases).

Serum antinuclear antibodies were detected in all patients: 4 anticentromere, 2 antinucleolar, 1 anti-Scl70, and 1 ANA speckled. Of interest, the positivity of ANA was observed in 4/6 LoS patients before the diagnosis of SSc.

No patient reported exposure to toxic substances or cigarettes smoke; autoimmune thyroiditis was a comorbidity in 2/8 patients.

Finally, no local treatment was employed for LoS, while low dosage of systemic steroids was administered.

## 4. Discussion

In the present study, we retrospectively evaluated a large cohort of SSc patients, in order to find the cases who presented also LoS; eight patients (2.4%) were found.

LoS and SSc are two distinct clinical entities that may share some features, such as the histopathological findings in the skin and the possible presence of antinuclear autoantibodies. In this perspective they might represent two ends of a unique disease spectrum [8].

LoS and SSc cannot be differentiated by histopathological examination because they share the same aspects: lymphocytic perivascular infiltration in the reticular dermis and swollen endothelial cells in the early phase, followed by thickened collagen bundles infiltrating the entire dermis and extending into subcutaneous fat in the late phase, with loss of eccrine glands and blood vessels, and “fat trapping.” Therefore, skin biopsy does not allow making differential diagnosis per se; conversely, the global evaluation of the clinical picture is fundamental for the diagnosis. LoS is characterized by the absence of sclerodactyly, RP, and nailfold capillary changes; moreover, even if patients with LoS commonly have nonspecific systemic symptoms, such as malaise, fatigue, arthralgias, and myalgias, as well as the presence of autoantibodies, the typical features of SSc visceral involvement are absent [3, 12].

Even if the course of LoS is usually benign, with slow resolution of the skin lesions, there are data in literature suggesting that LoS is not an exclusively cutaneous disease [13]. There is evidence of possible internal organs involvement and association with other connective tissue disease, and the evolution towards SSc was reported in pediatric population [8, 9].

In this study, we documented the close onset of both LoS and SSc in 3 patients and the apparent “evolution” from LoS to SSc in other 3 cases. Nonetheless, the appearance of LoS* after* SSc diagnosis (2 of our patients and others described in literature) raises the hypothesis of mere coexistence of LoS and SSc. The presence of RP and serum ANA positivity or typical videocapillaroscopic alterations can be considered “red flags” of SSc onset in patients with LoS, consistently with what is reported in the literature regarding pediatric population [8].

Interestingly, in our study, ANA positivity was reported in 4/6 individuals before the diagnosis of SSc. Otherwise, the presence of a scleroderma pattern at videocapillaroscopy was a useful finding for the formulation of SSc diagnosis [1, 11].

**Figure 1:**
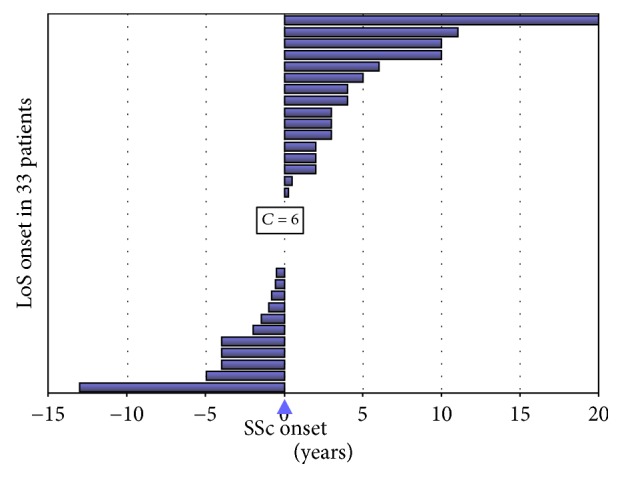
Graphical representation of the LoS onset in 33 patients (25 cases from the literature plus our 8 cases) concerning SSc onset (coloured bars correspond to the time spans between LoS and SSc beginnings). LoS may appear before or after SSc diagnosis, mainly in a time period between −5 and +5 years from SSc onset (27/33, 81.8%). To note, in 5 patients LoS and SSc presented contemporarily (“*C* = 6”).

The coexistence of SSc and LoS was already described in 3.2–6.7% of SSc patients [14–19]. Toki et al. [16] found 9 cases (M/F 3/6) of LoS out of 135 SSc patients, and 6 were ANA negative. In the study by Maricq [14] only 1 case out of 12 developed SSc 6 months after the onset of morphea, while the 2 diseases presented contemporary in other 4 patients; in all these cases the limited SSc subset was described. Chen et al. [15] described 8 patients with LoS out of 220 SSc case series, and in 3 patients LoS preceded the onset of SSc. Again, negative ANA were significantly prevalent in the overlap subjects. Interestingly, considering all the SSc/LoS cases described in the literature [14–19] plus our 8 patients, the LoS and SSc onsets are generally very close (mean LoS-SSc difference time: 1.5 ± 5.7 years; Figure 1). Indeed, the occurrence of two or more distinct autoimmune disorders suggest the presence of a common autoimmunity-prone background.

On the other hand, a prospective multicentre study performed in four French academic dermatology departments [20], including 76 patients with morphea and 101 age- and sex-matched controls, did not find predictive signs for SSc evolution in LoS patients, in comparison with controls. Indeed, the authors concluded that SSc and LoS are not likely as 2 entities belonging the same disease spectrum.

The main limit of our study is the small number of patients who presented both LoS and SSc. However, the coexistence of these 2 disorders seems to be quite rare; therefore it is difficult to recruit large case series. Therefore, the findings of this preliminary study should be confirmed in multicentre large cohort-based surveys.

In conclusion, LoS and SSc are 2 distinct clinical entities with autoimmune origin, and they are infrequently associated with each other. The possible onset of SSc in LoS patients should be considered, particularly in the cases that present features suggestive for SSc development, such as RP, presence of SSc-specific autoantibodies, or videocapillaroscopic abnormalities; in these cases, a careful clinical and laboratory follow-up is recommended.

## Figures and Tables

**Table 1:** Summary of the patients of our series with LoS associated with SSc.

Number	Age/sex	First diagnosis	Clinical picture at the 1st rheumatologic visit	Time to 2nd disease onset (months)	Second diagnosis	Clinical picture at the 2nd diagnosis	Other SSc features during the follow-up
(1)	26 F	LoS	Morphea at right leg from 2 years; RP onset 4 years before, new telangiectasias, nondiagnostic alterations at VC, ANoA with ENA neg.	24	SSc	RP, sclerodactyly and sclerodermic face, ACA plus anti-SSA, DU, “early” SSc pattern at VC	Esophagopathy

(2)	60 F	LoS	Morphea at the abdomen from 2 years, ANoA, nonspecific pattern at VC	48	SSc	ANoA, “active” SSc pattern at VC, Esophagopathy	RP, sclerodactyly and sclerodermic face, ILD

(3)	33 F	LoS	Recent onset of morphea at right arm and face, ANA speckled, SSc pattern at VC, 2 episodes of RP	7	SSc	RP, puffy hands, ANA speckled, “early” SSc pattern at VC	- *(pregnancy complicated by IUGR)*

(4)	69 F	LoS	Recent onset of morphea at dorsum, previous RP, puffy hands, Scl70, DU, “active” SSc pattern at VC	contemporary	SSc	-	Sclerodermic face

(5)	50 F	LoS	Recent onset of morphea at trunk and right thigh, doubtful very mild sclerodactyly, ANA speckled, aspecific pattern at VC	12	SSc	RP, sclerodactyly, ANoA, aspecific pattern at VC	Esophagopathy

(6)	83 F	LoS	Morphea at dorsum from 13 years, RP, ACA	156	SSc	RP, mild sclerodactyly, ACA, “early” SSc pattern at VC, sicca syndrome, DLCO 68%	DLCO further reduction (56%)

*(7)*	*70 F*	*SSc*	*RP, sclerodactyly, ACA, DU, Esophagopathy*	*120*	*LoS*	*Left pretibial linear LoS*	*-*

*(8)*	*50 F*	*SSc*	*RP, sclerodactyly, ACA, DU, “early” SSc pattern at VC, melanodermia, calcinosis*	*60*	*LoS*	*Morphea at trunk*	*-*

*Legend*. In the first 6 cases LoS was the first diagnosis made by a dermatologist; successively, these patients referred to ourRheumatology Unit because of the suspect of an unrecognized SSc. After a variable period, SSc diagnosis was formulated in presence of a SSc-specific clinical picture. During the follow-ups, eventual new features of the disease appeared; in the 7th and 8th case (italic rows) Los developed in the course of a definite SSc, in patients referring to our Rheumatology Unit. The second diagnosis (LoS) was confirmed by the dermatologist.
