# Erratum to “From Localized Scleroderma to Systemic Sclerosis: Coexistence or Possible Evolution”

**DOI:** 10.1155/2018/6984282

**Published:** 2018-06-05

**Authors:** Dilia Giuggioli, Michele Colaci, Emanuele Cocchiara, Amelia Spinella, Federica Lumetti, Clodovero Ferri

**Affiliations:** Scleroderma Unit, Chair of Rheumatology, University of Modena and Reggio Emilia, Modena, Italy

In the article titled “From Localized Scleroderma to Systemic Sclerosis: Coexistence or Possible Evolution” [1], the first and last names of all the authors were reversed. The revised authors' list is shown above.
